# A closer look at mammalian antiviral condensates

**DOI:** 10.1042/BST20231296

**Published:** 2024-05-23

**Authors:** J. Monty Watkins, James M. Burke

**Affiliations:** 1 Department of Molecular Medicine, The Herbert Wertheim University of Florida Scripps Institute for Biomedical Innovation and Technology, Jupiter, FL, U.S.A.; 2 Department of Immunology and Microbiology, The Herbert Wertheim University of Florida Scripps Institute for Biomedical Innovation and Technology, Jupiter, FL, U.S.A.; 3 Skaggs Graduate School of Chemical and Biological Sciences, The Scripps Research Institute, Jupiter, FL, U.S.A.

**Keywords:** condensate, G3BP1, PKR, RNA, RNase L, stress granules

## Abstract

Several biomolecular condensates assemble in mammalian cells in response to viral infection. The most studied of these are stress granules (SGs), which have been proposed to promote antiviral innate immune signaling pathways, including the RLR-MAVS, the protein kinase R (PKR), and the OAS-RNase L pathways. However, recent studies have demonstrated that SGs either negatively regulate or do not impact antiviral signaling. Instead, the SG-nucleating protein, G3BP1, may function to perturb viral RNA biology by condensing viral RNA into viral-aggregated RNA condensates, thus explaining why viruses often antagonize G3BP1 or hijack its RNA condensing function. However, a recently identified condensate, termed double-stranded RNA-induced foci, promotes the activation of the PKR and OAS-RNase L antiviral pathways. In addition, SG-like condensates known as an RNase L-induced bodies (RLBs) have been observed during many viral infections, including SARS-CoV-2 and several flaviviruses. RLBs may function in promoting decay of cellular and viral RNA, as well as promoting ribosome-associated signaling pathways. Herein, we review these recent advances in the field of antiviral biomolecular condensates, and we provide perspective on the role of canonical SGs and G3BP1 during the antiviral response.

## Introduction

The detection of viral replication at the earliest stage of infection is critical for the host to mount effective innate and adaptive immune responses required to clear the infection. Mammalian cells express pattern recognition receptor (PRRs) that bind to double-stranded RNA (dsRNA), which is commonly generated during viral replication [1–3]. PRR binding to dsRNA activates antiviral responses that repress viral replication, limit viral dissemination, and attract innate and adaptive cells to the site of infection. However, dysregulation of these pathways, such as viral-mediated attenuation or over-activation, can lead to viral-associated pathogenesis, cytokine release syndrome (cytokine storm), autoimmune disorders, neuroinflammation, and cancer [4–6].

Most mammalian cells contain three major antiviral pathways, which are the RLR-MAVS, protein kinase R (PKR), and OAS/RNase L pathways. In the RLR-MAVS pathway, RIG-I-like receptors (RLRs) (Rig-I or MDA-5) sense viral RNA in the cytosol and activate the mitochondrial antiviral signaling protein (MAVS), which is localized on the outer membrane of mitochondria, peroxisomes, and endoplasmic reticulum [7–13]. Activated MAVS leads to the phosphorylation of IRF3, which is a transcription factor that induces genes encoding for antiviral proteins, such as type I and III interferons, interferon induced proteins with tetratricopeptide repeat (IFIT) proteins, and PRRs [14]. PKR is activated upon binding dsRNA and shuts off canonical translation initiation by phosphorylating eIF2a on serine 51 [15]. This pathway has been reviewed both within the context of viral infection and more generally [16,17]. In the OAS-RNase L pathway, OAS protein recognizes dsRNA and produces 2′-5′-oligo(A), which activates RNase L by promoting homodimerization [18,19]. After activation, RNase L rapidly cleaves ssRNA regions in host and viral RNAs [19–29]. RNase L generally represses viral replication capacity by degrading host and viral RNA and by initiating inflammation and apoptosis [30,31].

How cells maintain the ability to rapidly activate innate immune pathways upon sensing viral replication, while also limiting their activation under homeostasis, is an important area of investigation. Biomolecular condensation has been implicated in regulating the sensing and activation of immune signaling pathways. Stress granules (SGs), which are host-generated biomolecular condensates that form as part of the integrated stress response (ISR), have long been implicated in regulating the activation and function of antiviral pathways. However, two recently identified biomolecular condensates, dsRNA-induced foci (dRIF) and RNase L-induced bodies (RLBs), complicate the simplified view of SGs being the primary regulator of antiviral signaling. Herein, we will review recent studies that represent a paradigm shift in our understanding of SGs, and we discuss the potential functions of dRIF and RLBs.

## SGs: antiviral signaling platforms or a consequence of antiviral signaling?

**Figure 1.**
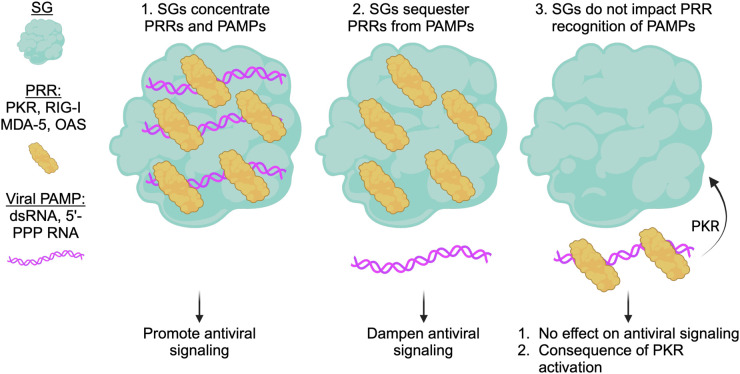
Potential roles for SGs in regulating antiviral signaling. Schematic of observed interactions between SGs (green), PRRs (yellow), and PAMPs (pink) and their effects on antiviral signaling. In model 1, SGs concentrate PAMPs and PRRs to promote antiviral signaling. In model 2, SGs sequester PRRs aways from PAMPs to dampen antiviral signaling. In model 3, SGs do not concentrate PRRs or PAMPs. SGs do not impact antiviral signaling, but instead are a consequence of antiviral signaling via the PKR pathway. Created with BioRender.com.

SGs have been widely modeled as platforms for innate immune activation in response to viral infection [32,33]. This is based on observations that SGs form during numerous viral infections, many viruses antagonize SG formation, antiviral proteins have been reported to localize to SGs, and knockout/knockdown of proteins required for SG assembly reduces the type I interferon response. Interactions between specific viruses and SGs have been extensively reviewed [32,33]. Here, we will review studies supporting three potential effects of SGs on antiviral signaling, whereby: (1) SGs promote antiviral signaling; (2) SGs dampen antiviral signaling; (3) SGs are incidental biomolecular condensates that are a consequence of antiviral signaling and do not regulate antiviral signaling (Figure 1).

### SG biogenesis and composition

SGs assemble in the cytoplasm when translation initiation is repressed via phosphorylation of eIF2α as part of the ISR, which is activated in response to several cellular stresses [34,35]. Viral-induced SGs typically assemble when PKR activates after binding viral dsRNA. PKR phosphorylates eIF2α on serine 51, which limits canonical translation initiation [15,36]. Once ribosomes run off mRNAs, the translationally stalled mRNAs condense into SGs.

While greater than 90% of cellular mRNAs undergo translational repression, only 9% of the mRNAs localize to SGs [37]. Mature SGs enrich hundreds of proteins and thousands of RNAs, including numerous RNA-binding proteins, long mRNAs, and long non-coding RNAs [37–39]. Critical proteins necessary for SG biogenesis include G3BP1/2, which act to condense and cross-link RNAs [40–42]. Long RNAs enrich in SGs due to their increased potential for multivalent interactions with other RNAs and RNA-binding proteins [43]. However, long RNAs are likely not the first RNAs to nucleate SGs due to the observation that SGs form prior to ribosomes running off long mRNAs based on the average rate of translation. Thus, SGs are likely nucleated with shorter RNAs, which may require G3BP1 and RNA modifications such as m6A [44,45].

While viral-induced SGs are only observed in a small percentage of infected cells under fixed conditions, live-cell experiments reveal they are transient and occur in a larger number of cells over the course of an infection. For example, SGs oscillate via a combination phosphorylation and dephosphorylation of eIF2α, both in acute and chronic infections [46,47].

### SGs as antiviral signaling platforms

Several studies have reported that antiviral proteins localize to SGs, including PKR, RIG-I, MDA-5, OAS1, OAS2, RNase L [33,48–51]. Thus, SGs have been proposed to promote the activation of the RLR-MAVS, PKR, or OAS-RNase L antiviral pathways by either concentrating PAMPs and PRRs in SGs, thus promoting PAMP recognition by PRRs, or by serving as a scaffold for antiviral signaling complexes to oligomerize [33,48–51] (Figure 1, model 1). A founding example of this model was reported during influenza A virus (IAV) PR8-ΔNS1 (H1N1) infection, whereby PKR-induced SGs were shown to concentrate Rig-I, PKR, dsRNA, and IAV RNAs [48]. Knockdown of either PKR or G3BP1 resulted in less interferon β mRNA induction in response to IAV/PR8-ΔNS1, thus leading to a model whereby PKR-mediated SG assembly results in Rig-I-MAVS-IRF3 signaling for IFNB mRNA induction. Moreover, SGs formed due to overexpression of G3BP1 were shown to trigger PKR-mediated phosphorylation of eIF2α [33]. While this was proposed to alter the RLR-MAVS pathway, it remains untested if it altered the OAS-RNase L pathway, which antagonizes IAV-Udorn/72-ΔNS1 (H3N2) replication [52].

### SGs as shock absorbers that dampen antiviral signaling

Consistent with previous studies, Paget et al. [53] recently reported that several cytoplasmic antiviral proteins (MAVS, RIG-I, MDA-5, OAS, RNase L, PKR) localize to SGs. However, Paget et al. proposed that SGs sequester PRRs and antiviral enzymes to dampen antiviral response (Figure 1, model 2). This is based on the observation that knockout of G3BP1/2 or PKR enhanced RLR-MAVS, PKR, and RNase L activity. It is important to note that the SGs observed in this study are likely RLBs as opposed to SGs. This is because both A549 and U-2 OS cells activate RNase L in response to poly(I:C), which inhibits SG assembly and promotes RLB assembly [21,54]. This is an important distinction because RLBs do not require G3BP1/2 or PKR for their assembly, so their observation that G3BP1/2-KO enhances antiviral signaling is inconsistent with their model that SGs (RLBs) sequester PRRs to dampen antiviral signaling. Moreover, the sequestration model proposed by Paget et al. [53] would require that a significant percentage of each individual antiviral protein be concentrated in SGs. Notably, only 18% of total G3BP1 localizes to SGs during stress, which is among the most enriched RNA-binding proteins in SGs [55]. Thus, it is unlikely that a significant percentage of antiviral proteins localize to SGs. Due to several inconsistencies with previous literature and studies discussed below, additional independent studies are needed to validate these findings. While G3BP1 could in principle dampen antiviral signaling in some contexts, it is uncertain if potential sequestration of antiviral proteins to either SGs or RLBs could account for this putative function of G3BP1.

### SGs are incidental and do not affect antiviral signaling

Several recent studies have demonstrated that SGs do not concentrate antiviral proteins to a degree that can alter antiviral signaling (Figure 1, model 3). First, two independent studies showed that PKR does not enrich in either sodium arsenite- or dsRNA-induced SGs (or RLBs) [56,57]. Notably, Corbet et al. observed this both via immunofluorescence assays controlled for PKR signal via PKR-KO cells and fluorescently tagged PKR. Similarly, neither RNase L nor OAS3 were observed to concentrate in either sodium arsenite-, dsRNA-, or viral-induced SGs (or RLBs) [58]. Lastly, RIG-I, MAVS, and IRF3 were not observed to concentrate in either SGs (or RLBs) induced by flavivirus infection or dsRNA lipofection [59]. The only PRR that has consistently been shown to localize to SGs is MDA-5 [50,59]. Interestingly, MDA-5 did not localize to RLBs, indicating that MDA-5 localization to SGs is specific. The mechanism by which MDA-5 localizes to SGs is unknown.

Consistent with SGs not concentrating antiviral proteins, our recent studies indicate that neither G3BP1 nor SGs regulate the activation or function of the PKR, RLR-MAVS-IRF3, or OAS-RNase L antiviral pathways [59]. Specifically, knockout of G3BP1/2 in RNase L-null cells, which abolished SG assembly, did not alter RLR-MAVS-IRF3-mediated signaling, induction of interferon β mRNA, or alterations to interferon β protein synthesis in response to dsRNA, 5′-PPP-RNA, or flavivirus infection [59]. Similarly, knockout of PKR in RNase L-null cells did not alter activation of RLR-MAVS-IRF3 signaling for interferon β mRNA induction in response to dsRNA or flavivirus infection. Thus, despite MDA-5 localizing to SGs [50], these data indicate that this does not affect MAVS activation, which is in agreement with previous findings by Langereis et al. [50]. Knockout of G3BP1/2 did not alter activation of PKR, based on phosphorylation of PKR or PKR-mediated phosphorylation of eFI2α. Lastly, knockout of G3BP1/2 in A549 cells did not alter RNase L-mediated rRNA cleavage or mRNA decay in response to dsRNA or infection with dengue virus serotype 2, West Nile virus, or Zika virus [59].

Taken together, these recent studies suggest that SGs do not concentrate enough antiviral proteins to modulate antiviral signaling to an observable degree, and that neither G3BP1 nor SGs generally promote or dampen antiviral signaling. However, we note that SGs could either enhance antiviral signaling in a context that may be specific to the virus or cell-type, or they could promote stress signaling pathways other than the RLR-MAVS, PKR, and OAS-RNase L antiviral pathways.

### Perspective on SGs and antiviral signaling

It is important that independent studies address the role of SGs in concentrating antiviral proteins and regulating antiviral signaling pathways considering the disparate findings described above. Nevertheless, how antiviral proteins such as dsRNA-binding PRRs (OAS3, PKR) and enzymes (RNase L) would conceivably concentrate in SGs, and how this impacts antiviral signaling, is uncertain considering the following observations:

- PRRs thought to localize to SGs typically bind dsRNA, but SGs concentrate ssRNA (mRNAs and long non-coding RNAs). Although SGs could generate dsRNA [60], perhaps via complementation of Alu elements within mRNAs, immunofluorescence assays demonstrated that SGs do not contain measurable levels of dsRNA [57,58]. Moreover, ADAR1, which localizes to SGs, may reduce dsRNA generated in SGs via A-I editing of dsRNA, thus limiting their ability to activate PKR and other dsRNA-binding PRRs [57,61].
- Because SGs form as a result of PKR-mediated phosphorylation of eIF2α, SGs cannot be required for the activation of PKR. Instead, SGs are the consequence of PKR activation. Although SGs generated by G3BP1 overexpression leads to PKR activation [33], it does not appear that canonical dsRNA-induced SGs function in the same capacity because G3BP1-null A549 cells activate similar levels of P-PKR and P-eIF2α [59]. It is possible that artificial overexpression of G3BP1 could promote dsRNA, leading to activation of PKR. Whether canonical SGs could function in this capacity or whether this alters PKR activity during viral infection is unclear. While Paget et al. propose that SGs negatively regulate PKR, cells already contain a negative feed-back loop to regulate PKR-mediated phosphorylation of eIF2α via GADD34, which dephosphorylates eIF2α to restore translation and disassemble SGs [62].
- SGs are not required for nor prevent the activation of RNase L because RNase L typically activates prior to PKR-mediated SG assembly [54]. Moreover, in cells that contain pre-formed SGs, RNase L can rapidly activate and lead to the disassembly of SGs [54].
- SGs are neither required nor prevent RLR-MAVS-signaling because RLR-MAVS-signaling often occurs in the absence of PKR-induced SGs. Moreover, RLR-MAVS-signaling is not inhibited in cells that contain PKR-induced SGs [21]. Lastly, RLR-MAVS-signaling is equivalent between parental, G3BP1/2-KO, or PKR-KO A549 cells in response to poly(I:C) lipofection or flavivirus infection [59].
- Many cell types, such as primary pulmonary artery endothelial cells and A549 lung carcinoma cells, activate RNase L prior to PKR and thus do not commonly assemble canonical SGs in response to dsRNA [58]. It is likely that SGs only form in cancer cell lines (Huh7, HEK293T) that do not activate RNase L nearly as frequently as the PKR pathway, or during viral infections that inhibit the OAS-RNase L pathway but not the PKR pathway.

These observations argue that cells likely do not use SGs as a primary means to activate or autoregulate antiviral signaling pathways. Conceptually, this is consistent with the fact that SGs are a product formed following the initiation of antiviral signaling. Because G3BP1 and PKR have SG-independent functions, future studies should exercise caution in attributing their putative function in regulating antiviral signaling on their ability to promote SG assembly.

### Paracrine granules

Paracrine granules (PGs) are form in uninfected cells as a result of paracrine signaling from virally infected cells [63]. The ability of cells that have yet-to-be infected by a virus to form PGs from paracrine signals represents a potential novel mechanism by which SGs could function, whereby their formation prior to viral infection could directly alter the early phase of viral infection or alter antiviral signaling. Further studies are needed to determine how PGs broadly impact the antiviral response.

## Viral-aggregated RNA condensates

Despite evidence that neither G3BP1 nor SGs alter antiviral signaling [50,59], several observations suggest that G3BP1 can antagonize specific viruses [33], whereas others utilize G3BP1 to promote replication [64]. The pro- and anti-viral functions of G3BP1 have been recently reviewed [65]. Here, we will focus on recent findings that suggest that G3BP1 can condense viral RNA as part of an intrinsic innate immune defense mechanism.

The SARS-CoV-2 nucleocapsid (N) protein binds G3BP1, which prevents SG assembly during SARS-CoV-2 infection, even when an exogenous SG-inducing stimulant such as sodium arsenite is applied to infected cells and increases p-eIF2α [59,66–68]. Canonical SGs do not form during SARS-CoV-2 infection because SARS-CoV-2 Nsp1 and RNase L degrade host mRNA during SARS-CoV-2 infection [67,68]. However, the activation of RNase L results in RLB assembly during SARS-CoV-2 infection. Notably, G3BP1 does not incorporate into RLBs due to it inhibition by SARS-CoV-2 N protein [68].

A primary question in the field is: *why does SARS-CoV-2 N protein interact with G3BP1 if SGs cannot form during SARS-CoV-2 infection?* Although G3BP1 could be a host factor required for SARS-CoV-2 replication, similar to Norovirus [64], SARS-CoV-2 replicated to similar titers in parental and G3BP1/2-KO A549 cells [59]. This suggests that the interaction between G3BP1 and N was not required for SARS-CoV-2 replication.

Importantly, a mutant of G3BP1 (F124W) that abolishes SARS-CoV-2 N protein interaction with G3BP1 resulted in condensation of G3BP1 and SARS-CoV-2 RNA into SG-like aggregates during SARS-CoV-2 infection [59,66]. Notably, these G3BP1-viral RNA aggregates lack host mRNAs due to the decay of host mRNAs by SARS-CoV-2 Nsp1 and the activation of RNase L [68], thus we termed these viral aggregated RNA condensates (VARCs) (Figure 3) [59]. VARC assembly correlates with lower viral RNA levels, lower viral translation output, and reduced viral output. However, VARC assembly was rare despite the presence of p-eIF2α in most SARS-CoV-2 infected cells. An important question to address is whether VARCs undergo assembly and disassembly, similar to SGs [46,47], which could suggest that many more SARS-CoV-2-infected cells assemble VARCs during infection.

The assembly of VARCs was promoted by inhibition of eIF4 translation initiation and RNA helicase functions via pateamine A or hippuristanol treatment, respectively. Thus, translational repression of SARS-CoV-2 RNA combined with the G3BP1-mediated RNA condensing activity can lead to condensation of viral RNA into an SG-like state that perturbs viral RNA functions, such as translation, replication, and packaging.

Unlike SARS-CoV-2, flaviviruses such as dengue virus, Zika virus, and West Nile virus do not inhibit G3BP1. Thus, PKR activation leads to SG assembly as a result of phosphorylation of eIF2α. However, the full-length genomic RNAs of these viruses do not accumulate in SGs, even when p-eIF2α levels are increased via sodium arsenite treatment [59]. These data indicate that flavivirus RNAs avoid being condensed in SGs due to their ability to translate in the presence of p-eIF2α at later times post-infection. Consistent with this, inhibition of the translation and helicase activity of eIF4A via pateamine A or hippuristanol treatment, respectively, resulted in robust accumulation of West Nile virus and Zika virus RNA genomes/mRNAs in SGs.

Collectively, these data indicate that viruses use translation, eIF4A RNA de-condensor activity, and inhibition of G3BP1 to prevent their condensation by G3BP1 [59]. Viruses with long genomes, such as SARS-CoV-2 (∼29 kb), are likely prone to G3BP1-mediated RNA condensation due to higher potential for forming multivalent interactions, similar to long mRNAs highly enriching in SGs [37]. Notably, the observation that flavivirus RNAs can incorporate into SGs upon inhibition of their translation initiation is similar to the observation that IAV-PR8-ΔNS1 mRNAs accumulate in SGs [48]. However, because IAV mRNAs are short (less than 2.5 kb, which is ∼ the average length of human mRNAs), whereas flaviviruses are longer (∼10.5 kb), an important question to address is what percentage of IAV mRNAs accumulate in SGs.

**Figure 2.**
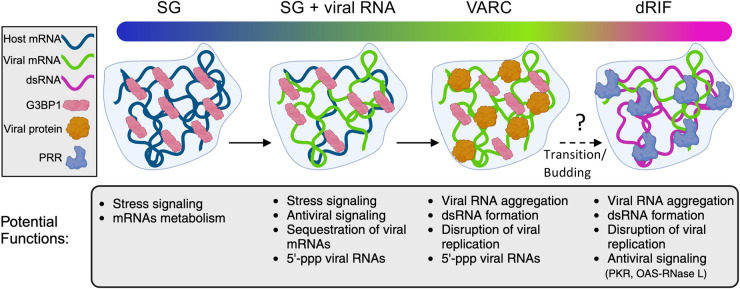
Viral-aggregated RNA condensates. During infections that degrade host mRNAs, such as SARS-CoV-2 infection, G3BP1 can condense viral RNAs, leading to VARCs, which may disrupt viral replication by interfering with the viral replication organelle. Created with BioRender.com.

Pateamine A- or hippuristanol-induced SGs containing flavivirus RNAs did not contain dsRNA, though large dsRNA structures were adjacent to these SGs [59]. In contrast, SGs assembled during IAV-PR8-ΔNS1 infection stained of dsRNA [48], similar to VARCs observed during SARS-CoV-2 infection. Studies are under way to determine if the incorporation of IAV and flavivirus RNA into SGs alters antiviral signaling or viral replication. Lastly, the lack of host mRNAs in VARCs may fundamentally alter the ability of G3BP1 to initiate antiviral signaling. Moreover, unlike SGs, VARCs can contain dsRNA and thus could potentially serve as or seed structures, such as dRIF (discussed below), which could serve as antiviral signaling platforms (Figure 2). Future work will address if VARCs can promote PRR recognition of dsRNA in VARCs.

## Double-stranded RNA-induced foci

**Figure 3.**
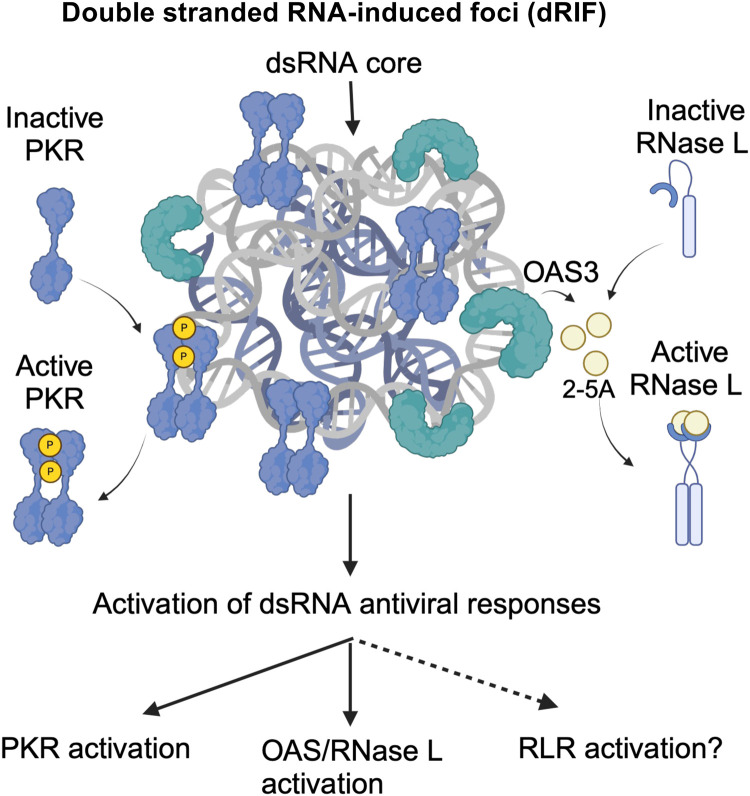
Double-stranded RNA-induced foci regulate the initiation of antiviral signaling. Double stranded RNA-induced foci (dRIF) concentrate dsRNA-binding proteins including PKR, OAS3, and ADAR1. In addition, antiviral effectors such as RNase L also concentrate at dRIF. The assembly of dRIF correlates with the activation of the PKR and OAS-RNase L pathways. Created with BioRender.com.

The lack of robust evidence supporting that SGs directly regulate antiviral signaling questions whether biological condensation plays a role promoting antiviral signaling. However, a condensate termed dRIF was recently shown to concentrate dsRNA and dsRNA-binding proteins such as PKR in the cytoplasm (Figure 3) [57]. While the biogenesis mechanisms of dRIF remain uncharacterized, dRIF have been observed following the lipofection of electroporation of dsRNA into cells [53,56–58]. Evidence that dRIF form during viral infection is that small percentage of measles-infected cells that displayed PKR puncta [56]. Moreover, puncta that co-stain for viral dsRNA, P-PKR, OAS3, and RNase L were observed during Dengue virus, West Nile virus, and Zika virus infection [58].

The role of dRIF in regulating innate immune signaling is mostly uncharacterized, though they are implicated in regulating the activity of PKR [56–58]. Whereas Zappa et al. propose that PKR concentration at dRIF is inhibitory for PKR functions, Corbet et al. present evidence that PKR concentration at dRIF correlates with PKR-mediated translation repression. Further supporting that dRIF promote the initiation of antiviral signaling, concentration of OAS3 and RNase L at dRIF correlates with RNase L activation [58].

The composition of cellular proteins that localize to dRIF has not been comprehensively characterized. The identification of dRIF highlights the need for comprehensive studies on the localization of other dsRNA sensors, such as the RLR family and additional OAS-family proteins during viral infections. Studies addressing if dRIF are necessary for innate immune activation, which viral infections cause dRIF assembly, if and how viruses combat dRIF assembly are underway.

## RNase L-induced bodies

**Figure 4.**
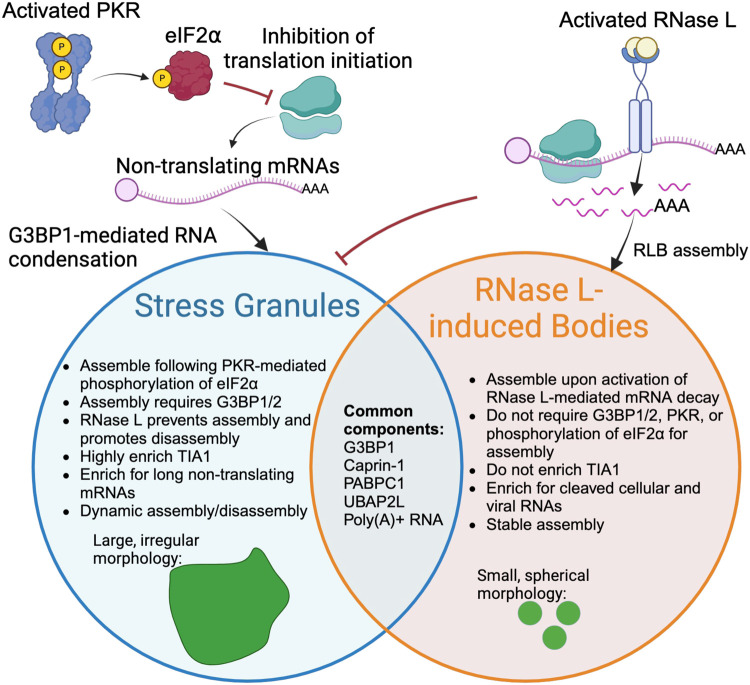
Relationship between RNase L-induced bodies and stress granules. Schematic of the SG and RLB assembly biogenesis pathways are displayed above a Venn diagram that illustrates the differences and similarities between the biogenesis, composition, and morphology of SGs and RLBs. Created with BioRender.com

An RLB is a biomolecular condensate that assembles upon the initiation of RNase L-mediated mRNA decay [21,54,69]. This is based on the observation that RLB assembly is dependent on the ability of RNase L to cleave RNAs, and that RLBs are not observed in cells with intact host mRNA [21]. RLBs are similar to SGs in that they enrich some SG-associated RNA-binding proteins (G3BP1, Caprin 1, PABPC1) and poly(A)+RNA (Figure 4). However, RLBs differ from SGs based on several observations. First, unlike SGs, RLBs are not dependent on PKR-mediated of phosphorylation of eIF2α and ribosome run-off of mRNAs, as RLBs form in PKR-KO cells, in MEF- eIF2α-S51A cells, and in the presence of cycloheximide [54]. In addition, RLBs do not require G3BP1-mediated RNA condensation for their formation because they can assemble in G3BP1/2-KO cells [21,54,70]. Second, whereas SGs are large and irregularly shaped, RLBs are invariably small and spherical. Third, some SG-associated proteins, such as TIA1, do not enrich in RLBs. Lastly, whereas SGs accumulate long RNAs, intact mRNAs are not typically enriched in RLBs despite enrichment of poly(A)+RNA [54], suggesting that RLBs contain RNA cleavage products of RNase L.

Consistent with idea that RLBs concentrate cleaved RNA, RLBs were shown to sequester subgenomic flavivirus RNA (sfRNA) during infection with Dengue virus, Zika virus, or West Nile virus [71]. sfRNAs are stable 3′-end fragments of the flavivirus genome that are known to inhibit host RNA decay via binding to the host 5′-3′ exoribonuclease, XRN1 [72–74]. Prior to RNase L activation, sfRNAs distribute throughout the cytoplasm or interact with P bodies [71], which enrich for XRN1 and mRNA decay machinery [75,76]. However, upon RLB assembly due to RNase L activation, sfRNAs re-localize into RLBs, and host cellular decay machinery can degrade viral RNA [71].

**Figure 5.**
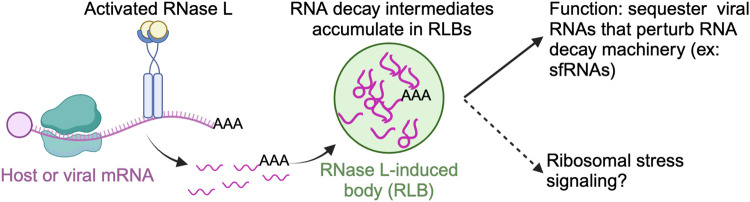
RNase L-induced bodies concentrate RNase L-cleaved host and viral RNA. Activation of the OAS-RNase L pathway leads to RNase L cleaving host mRNAs and viral genomes/mRNAs. The cleavage fragments, particularly 3′-end fragments, concentrate into RNase L-induced bodies (RLBs). The sequestration subgenomic flavivirus RNAs (sfRNA) reduces the ability of sfRNAs to inhibit mRNA decay machinery in P-bodies. Because RNase L cleavage of RNA leads to phosphorylation of eIF2α, RLBs may be a sight of ribosome-mediated stress responses. Created with BioRender.com.

Based on these observations, we propose a several possible functions of RLBs (Figure 5). Firstly, RLBs may sequester RNAs that cellular RNA decay machinery cannot fully degrade. This is supported by the fact that poly(A)+RNA but not full-length mRNAs highly enrich in RLBs [54]. A second possibility is that RNase L cleavage directly leads to RNA incorporation into RLBs. In this model, RLBs may act as sites of further decay, and primarily reflect the growing number of cleaved RNAs in the cell. Lastly, the accumulation of cleaved mRNAs in RLBs suggests that RLBs may be platforms for signaling for ribosome-mediated stress responses.

Identification of specific RNAs localized in RLBs is an important next step for understanding these RNP granules. Additionally, a more thorough understanding of the protein content, particularly identification of markers unique from SG markers, would greatly benefit the field's ability to study these granules. Finally, because there is currently no method of knocking out RLBs other than abolishing the ability of RNase L to degrade RNA [21], screens that identify proteins that are potentially required for RLB assembly other than RNase L are paramount.

## Perspectives

- Biomolecular condensates, including SGs, RLB, and dRIF, can form during viral infection and may regulate the activation and function of antiviral pathways.
- SGs have been predominantly thought to promote antiviral signaling. However, the field is shifting away from this model due to the observation that antiviral proteins do not substantially enrich in SGs, neither G3BP1 or SGs modulate antiviral signaling, SGs do not form in cells that have activated RNase L, and antiviral proteins localize to dRIF instead of SGs. Despite this shifting paradigm, determining the function of SGs during viral infection remains paramount.
- Future studies will need to address the composition and biogenesis of RLB and dRIF, how they impact the antiviral response, and how viruses antagonize them.

## Open Access

Open access for this article was enabled by the participation of University of Florida in an all-inclusive *Read & Publish* agreement with Portland Press and the Biochemical Society under a transformative agreement with Individual.

## Abbreviations

dRIF: double-stranded RNA-induced foci
dsRNA: double-stranded RNA
IAV: influenza A virus
ISR: integrated stress response
MAVS: mitochondrial antiviral-signaling
OAS: oligoadenylate synthetase
PG: paracrine granule
PKR: protein kinase R
PRR: pattern recognition receptor
RLB: RNase L-induced body
RLR: RIG-I-like receptor
RNase L: ribonuclease L
sfRNA: subgenomic flavivirus RNA
SG: stress granule
VARC: viral aggregated RNA condensate
